# Editorial: Survival strategies: cellular responses to stress and damage

**DOI:** 10.3389/fcell.2025.1630652

**Published:** 2025-06-19

**Authors:** Bhawana Bissa, Aurore Claude-Taupin, Pavel Ivanov, Jingyue Jia, Jing Pu

**Affiliations:** ^1^ Department of Biochemistry, School of Life Sciences, Central University of Rajasthan, Ajmer, India; ^2^ Université Paris Cité, INSERM U1151, CNRS U8253, Institut Necker-Enfants Malades, Paris, France; ^3^ Department of Medicine, Brigham and Women’s Hospital and Harvard Medical School, HMS Initiative for RNA Medicine, Boston, MA, United States; ^4^ Center for Global Health, Department of Internal Medicine, University of New Mexico Health Sciences Center, Albuquerque, NM, United States; ^5^ Autophagy, Inflammation and Metabolism Center of Biochemical Research Excellence, Albuquerque, NM, United States; ^6^ Department of Molecular Genetics and Microbiology, University of New Mexico Health Sciences Center, Albuquerque, NM, United States

**Keywords:** stress granules (SG), autophagy, metabolism, oxidative stress, cell signaling, membrane damage

Stress and damage in organisms originate at the cellular level and require immediate response to prevent onset of pathological conditions. The ability of cells to effectively counteract stress is vital for the overall health and survival of the organism. The stress that cells encounter varies, including oxidative stress, genotoxic stress, heat shock, nutrient deprivation, hypoxia, exposure to toxic compounds, pathogen invasion, mechanical injury and aging (Fulda et al., 2010). These stress conditions disrupt cellular homeostasis by damaging structural components or impairing critical functions. The ability of cells to offset and withstand stress conditions is primarily mediated by activation of survival pathways such as DNA damage response (DDR), heat shock response (HSR), unfolded protein response (UPR), anti-oxidant response, mitochondrial stress signaling, stress granules (SGs) and autophagy (Yan et al., 2021; Bahar et al., 2016). Beyond these pathways, inter-organellar crosstalk plays a crucial role in mitigating stress conditions (Raimundo, 2014). Furthermore, AMPK and mTOR play essential roles in managing cellular stress by coordinating their actions to restore cellular homeostasis (Hardie, 2015; Saxton and Sabatini, 2017). Depending on the level and type of stress, different pro-survival mechanisms are activated; however if the cell fails to neutralize the stress, various cell death pathways are initiated and eventually lead to disease states such as diabetes, neurodegeneration, cancer, and others (Fulda et al., 2010; Martindale and Holbrook, 2002).

The crosstalk between different survival pathways is important to preserve cellular functions and to repair macromolecular damage. For example, DDR and UPR direct a coordinated response, especially under hypoxic conditions to maintain cell integrity and prevent cell death (Endoplasmic Reticulum Stress and Unfolded Protein Response, 2024). Similarly, HSR can assist UPR to relieve ER stress by enhancing ER export of misfolded proteins (Kim and Gross, 2013). This coordination is essential when UPR is overwhelmed as in case of ER stress–mediated diabetes (Eizirik et al., 2008). Further, to combat oxidative stress and maintain redox homeostasis, cells utilize robust antioxidant response such as the Nuclear Factor Erythroid 2-Related Factor 2 (Nrf2)/Kelch-like ECH-associated Protein 1 (Keap1) signaling pathway and mitochondrial antioxidant systems to prevent molecular damage (Ma, 2013; Dodson et al., 2019). Various cellular stressors can induce the formation of SGs, which are cytoplasmic biomolecular condensates containing RNA and proteins (Hofmann et al., 2021). SGs can be selectively cleared by autophagy, a process termed granulophagy, to restore cellular homeostasis (Buchan and Parker, 2009). Moreover, autophagy acts as a survival mechanism under nutrient deprivation and helps remove damaged or dysfunctional cellular components to restore metabolic balance (Mizushima and Komatsu, 2011).

Thus, mammalian cells have developed an intricate and robust network of survival pathways that allow adaptation to different stress conditions. Intracellular responses to stress, their regulation and their pathophysiological implications have been extensively studied. This collection is an attempt to bring together recent studies on the stress response mechanisms highlighting different aspects of survival strategies, ranging from strategies of maintaining lysosomal homeostasis to responses to oxidative damage.

The collection includes 5 review articles, 2 original articles and 2 method articles. Wang et al. reviewed the cellular response to lysosomal damage, including alternative secretion pathways and interactions with other organelles from the endolysosomal system, ER and Golgi apparatus. They also explored the contribution of these processes to disease progression. Lysosomal damage responses also involve the conjugation of ATG8 (Autophagy-related protein 8) to single membranes (CASM), as reviewed by Kaur et al. The authors summarized how the decoration of lysosomes with ATG8 promotes downstream effects, such as membrane repair and removal.

Similar to the lysosomal membrane, the nuclear envelope can undergo rupture. In this collection, Di Bona and Bakhoum provided a detailed protocol to study real-time nuclear and micronuclear rupture and repair. They introduce a novel high-resolution fluorescence microscopy-based technique that allows for the induction and imaging of both micronuclear and primary nuclear damage.

In cancer cells, DDR is often dysregulated. Ghai et al. reviewed the roles of Heat Shock Factor 1 (HSF1) in cancer chemoresistance, including through the inhibition of the DDR pathway, thus promoting genomic instability and carcinogenesis. They also highlighted HSF1’s role in apoptosis inhibition and autophagy activation, and discussed the therapeutic potential of HSF1 inhibitors to overcome chemoresistance.

As in cancer, autophagy dysregulation has also been implicated in Parkinson’s disease. An original study by Limanaqi et al. explored the impact of alpha-synuclein - whose abnormal forms have been linked to Parkinson’s disease - on THP1 monocytes and derived macrophages. The authors showed that alpha-synuclein exerted cell- and context-specific effects, including alterations in autophagy, lipid dynamics, and inflammatory pathways, which may help explain the functional impairments of monocytes and macrophages observed in this neurological disorder.

Mitochondrial dysfunction is linked to numerous pathologies. Jiménez-Loygorri et al. described a method to quantitate mitophagy (a selective type of autophagy targeting mitochondria) flux, using the mitoQC reporter and flow cytometry. This approach can be multiplexed with the analysis of other cellular parameters, such as ROS production and cell viability in cultured cells, and has also been validated *ex vivo* in the retina of mitoQC reporter mice.

Sergiev et al. described the role of the mitochondrial protein Mitoregulin in regulating mitochondrial function. They summarized the literature–including sometimes, contradictory findings–on mitoregulin’s role in cellular metabolism, particularly in kidney and muscle physiology.

Oxidative stress, characterized by the accumulation of reactive oxygen species (ROS), is another major driver of cellular damage. Liu et al. reviewed how radiotherapy induces skin reactions, notably through oxidative stress, and discussed how antioxidant therapies could help prevent these side effects, thus improving cancer patients’ quality of life after radiotherapy. Extending the focus on strategies to protect cells from stress-related damage, Li et al. described a novel method to improve hepatocyte survival upon cryopreservation, using ultrasonic ice seeding technology.

Altogether, this Research Topic gathers original articles, reviews, and methodological papers that explore various aspects of cellular responses to stress and damage, to promote cell survival. We hope it will become a valuable resource for the current and the broader field.
