# Correction: Editorial: Survival strategies: cellular responses to stress and damage

**DOI:** 10.3389/fcell.2025.1665855

**Published:** 2025-09-11

**Authors:** Bhawana Bissa, Aurore Claude-Taupin, Pavel Ivanov, Jingyue Jia, Jing Pu

**Affiliations:** ^1^ Department of Biochemistry, School of Life Sciences, Central University of Rajasthan, Ajmer, India; ^2^ Université Paris Cité, INSERM U1151, CNRS U8253, Institut Necker-Enfants Malades, Paris, France; ^3^ Department of Medicine, Brigham and Women’s Hospital and Harvard Medical School, HMS Initiative for RNA Medicine, Boston, MA, United States; ^4^ Center for Global Health, Department of Internal Medicine, University of New Mexico Health Sciences Center, Albuquerque, NM, United States; ^5^ Autophagy, Inflammation and Metabolism Center of Biochemical Research Excellence, Albuquerque, NM, United States; ^6^ Department of Molecular Genetics and Microbiology, University of New Mexico Health Sciences Center, Albuquerque, NM, United States

**Keywords:** stress granules (SG), autophagy, metabolism, oxidative stress, cell signaling, membrane damage

The funder NIH NIGMS (GM126150 and GM146997) to Pavel Ivanov was erroneously omitted.

The original article has been updated.

